# Artifacts in fluoroscopy and changes in radiation dose caused by heating blankets and insulating covers during simulated endovascular treatment

**DOI:** 10.1007/s10140-020-01798-x

**Published:** 2020-05-30

**Authors:** Paweł Podsiadło, Grzegorz Liszka, Tadeusz Popiela, Tomasz Sanak, Sylweriusz Kosiński, Tomasz Darocha

**Affiliations:** 1 Department of Emergency Medicine, Jan Kochanowski University, IX Wieków Kielc 19, 25-317 Kielce, Poland; 2 Laboratory of Radiological Measurements GL Center Ltd., Tychy, Poland; 3 Department of Radiology, Jagiellonian University Medical College, Kraków, Poland; 4 Department of Disaster Medicine and Emergency Care, Jagiellonian University Medical College, Kraków, Poland; 5 Faculty of Health Sciences, Jagiellonian University Medical College, Kraków, Poland; 6 Department of Anesthesiology and Intensive Care, Medical University of Silesia, Katowice, Poland

**Keywords:** Hypothermia, Fluoroscopy, Radiation exposure, Endovascular procedures, Trauma

## Abstract

**Purpose:**

We aimed to assess whether insulating covers and warming systems cause artifacts in fluoroscopy, and whether they alter the radiation dose.

**Methods:**

Eight insulating and warming systems were wrapped around the phantom in order to obtain images in fluoroscopy, and to measure the absorbed and scattered radiation dose. A dosimeter, endovascular catheters, and stents were placed into a phantom. The other dosimeter was placed outside of a C-arm table, at the operator’s and anesthesiologist’s locations.

**Results:**

Most of the insulating covers did not cause artifacts in the fluoroscopy and led to a significant decrease in both the absorbed and scattered radiation dose. The highest decrease in the absorbed dose was observed with metalized foil (− 2.09%; *p* = 0.001) and in the scattered dose with Helios cover (− 55%; *p* < 0.001). Only one heating system (Ready Heat combined with Hypothermia Prevention and Management Kit cover) caused significant artifacts and increased radiation up to 99% (*p* < 0.001).

**Conclusion:**

Thermal insulation may be maintained during X-ray-guided emergency endovascular procedures in trauma victims. Self-heating blankets should be replaced with another warming system.

## Introduction

Post-traumatic hypothermia is a well-known risk factor for mortality in trauma victims [1]. The drop in body temperature is associated with clotting disorders, increased blood loss, organ failure, and worse outcome [2, 3]. Victims of severe trauma, e.g., with traumatic aortic injury (TAI), are often hypothermic on admission to hospital and remain hypothermic until the end of surgery [4]. Endovascular treatment of aortic injuries is linked to lower transfusion requirements and a lower risk of hypothermia when compared with traditional “open” surgery [4]. Nonetheless, among patients undergoing endovascular brain aneurysm treatment, more than half became hypothermic (< 36 °C) after 40 min, and all of them after 2 hours [5]. An additional cooling factor is the temperature in an angiography suite which usually does not exceed 20 °C, probably for the thermal comfort of the medical staff that wear X-ray protective aprons apart from surgical uniforms [5, 6]. Finally, anesthesia or sedation blunts the thermoregulatory response and increases the patient’s susceptibility to cold [7]. In a study by Khoynezhad et al., the mean Injury Severity Score in patients suffering from TAI was 38; pelvic fractures coexisted in 40%, and unstable spine fractures in 14% of patients [8]. Regarding the aforementioned circumstances and comorbidities, patients with TAI should remain on the backboard, while the thermal insulation which has already been applied pre-hospitally should be maintained. According to the Advanced Trauma Life Support recommendations, continuous prevention of hypothermia with blankets and warming devices is mandatory in trauma patients [9]. This may be provided by the passive warming (insulation) or devices which actively deliver the heat. Nevertheless, some doubts persist as to the potential of causing artifacts by certain materials used for heat balance improvement. While this has been studied on CT [10], no study assessed radiation changes and artifacts on fluoroscopy.

The aim of our study was to assess whether insulating covers hinder the visibility of endovascular devices in fluoroscopy and whether they cause changes in radiation doses absorbed by the patient and by medical personnel.

## Methods

### Study design

A prospective experimental study was conducted. A cubic phantom of 20-cm thickness was built using polymethyl methacrylate (PMMA) parts and placed on an angiographic X-ray system (Siemens Artis Zee), since a professional CTDI phantom built of PMMA could not be used due to another type of dosimeter being required. Endovascular devices, such as catheters and stents, and a Piranha R100 dosimeter (RTI Electronics) were placed into the central zone of our phantom. Automated C-arm settings were used, namely DSA/Body/Abdomen in FLAngio protocol; kVp was 67.7; magnification normal; source-to-image receptor distance (SID) was 100 cm. Initially, dose measurements during a fluoroscopy lasting 10 s were obtained three times to check repeatability and in this way, the precision of the applied methods. Then, the phantom was placed on a spineboard (Iron Duck, Chicopee, MA, USA) without any wrapping and measurements were repeated to obtain the reference values. Radiographic images were recorded. Subsequently, the phantom lying on the spineboard was wrapped and/or covered using the following materials:

1. A Blizzard Survival Blanket (Blizzard Protection Systems Ltd., Bethesda, UK),
2. A Hypothermia Prevention and Management Kit – HPMK (North American Rescue, Greer, S.C., USA) + polyester blanket + Ready Heat heating blanket (Tech Trade, Jersey City, NJ, USA),
3. HPMK + polyester blanket
4. LESS Thermal Bag (Less AS, Kapp, Norway)
5. Warm Touch – forced air warming blanket (Covidien, Mansfield, MA, USA)
6. Helios cover (TacMed Solutions, Anderson, SC, USA)
7. Ultrathin metalized foil (MF) + polyester blanket + ultrathin metalized foil (three-layer cover)
8. Mediwrap (Medical Innovations Group, Shoeburyness, Essex, UK)

Ready Heat (no. 2) and Warm Touch (no. 5) are warming devices, and remaining items are insulating systems.

### Radiation dose assessment

**Fig. 1:**
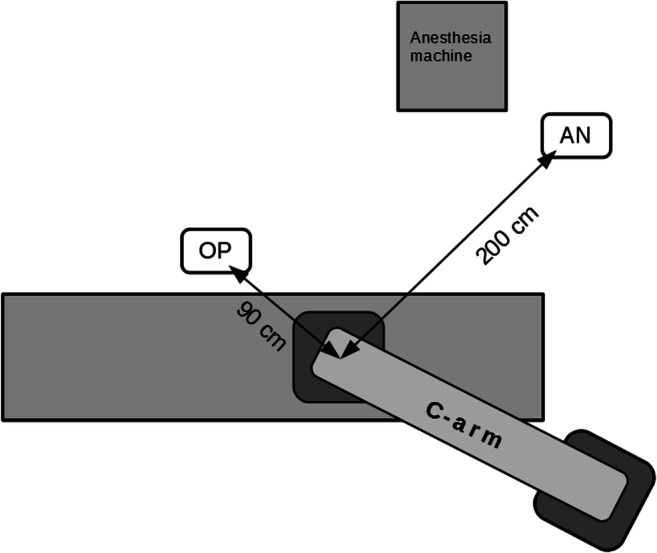
Places of measurement of the scattered dose (OP, operator; AN, anesthesiologist)

Measurements were obtained three times in every wrapping. Radiation values from the phantom dosimeter were recorded during 10 s of fluoroscopy with a frame rate of 7.5 fps. Dedicated software, namely Ocean 2014 Professional (RTI Electronics), calculated the dose absorbed per each frame. Simultaneously, scattered radiation was measured with an RK 100 dosimeter (Polon SA) placed in the operator’s and anesthesiologist’s locations. The distance from the X-ray axis to the operator’s place was 90 cm and to the anesthesiologist’s place was 200 cm (Fig. 1). No additional X-ray protective screens were used. Scattered radiation was recorded within 10 s and expressed in mcSv/h. Finally, the spineboard was removed and radiation measurements were repeated without any wrapping.

### Image quality assessment

**Fig. 2:**
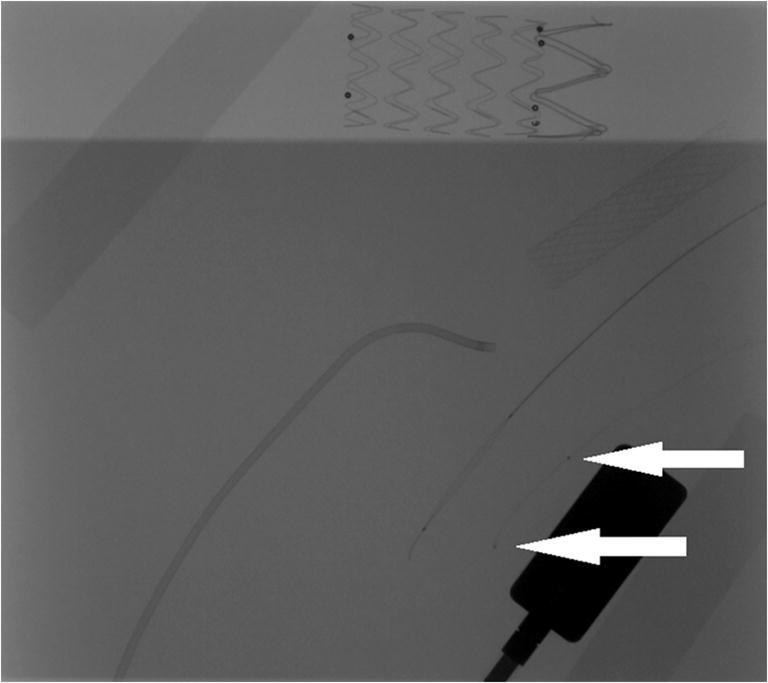
The 0.56-mm markers used to assess image resolution (arrows)

Three physicians (A, B, and C) with experience in X-ray-guided endovascular procedures assessed all images independently. To make this evaluation uniform, we have defined artifacts as every additional element visible in the picture which has been caused by insulating or warming covers. Artifacts were described as S—significant (hindering stent positioning, making catheter markers invisible), M—minor (visible but not hindering the procedure), and N—none. Image resolution was deemed not worsened if the markers of a 1.7F (0.56 mm diameter) microcatheter remained visible. Figure 2 was the reference picture, since it was taken without any wrapping.

### Statistics

Radiation doses, due to their normal distribution, were presented as mean and standard deviation. Data were compared with reference values and calculated using the Student *t* test. Statistical significance was defined as *p* < 0.05.

## Results

### Radiation dose assessment

A decrease in the absorbed dose was observed in the majority of tested covers. The highest decrease was caused by a three-layer wrapping (MF + blanket + MF), which was − 2.09% (*p* = 0.001). The Ready Heat blanket, integrated with the HPMK cover, increased the absorbed dose significantly up to 53.71% (*p* < 0.001).

Scattered dose values were higher in the operator’s location than those in the anesthesiologist’s. However, the proportion of doses in these two places was different for every cover. Most covers caused a decrease in the scattered dose apart from the Ready Heat blanket which increased radiation by 13% for the anesthesiologist and 99% for the operator (*p* = 0.003 and *p* < 0.001, respectively).

Complete data are shown in Table 1.

**Table 1:** Artifacts in fluoroscopy and radiation dose alteration caused by insulating and warming covers

	Artifact assessment			Radiation dose assessment								
				Absorbed dose (mcGy/frame)			Scattered dose (mcSv/h)					
	A	B	C	Patient	Dose change	*p*	Anesthesiologist location	Dose change	*p*	First operator location	Dose change	*p*
No insulation	N	N	N	1.3435 ± 0.0047	-	-	25.80 ± 1.35	-	-	138.67 ± 4.51	-	-
Blizzard	N	N	N	1.3320 ± 0.0001	− 0.86%	0.013	22.43 ± 1.39	− 13%	0.039	79.20 ± 1.23	− 43%	< 0.001
HPMK + Ready Heat	S	S	S	2.0651 ± 0.0086	53.71%	< 0.001	51.27 ± 2.78	99%	< 0.001	157.00 ± 2.00	13%	0.003
HPMK	N	N	N	1.3368 ± 0.0049	− 0.50%	0.162	21.47 ± 2.90	− 17%	0.079	98.80 ± 0.35	− 29%	< 0.001
LESS	M	M	M	1.3407 ± 0.0067	− 0.21%	0.581	20.07 ± 1.57	− 22%	0.009	68.83 ± 2.76	− 50%	< 0.001
Warm Touch	N	N	N	1.3225 ± 0.0036	− 1.56%	0.004	16.43 ± 2.23	− 36%	0.003	70.83 ± 6.51	− 49%	< 0.001
Helios	M	M	M	1.3556 ± 0.0040	− 0.90%	0.027	27.67 ± 1.72	7%	0.213	62.30 ± 2.95	− 55%	< 0.001
MF + blanket + MF	N	N	N	1.3154 ± 0.0040	− 2.09%	0.001	23.83 ± 3.16	− 8%	0.377	97.50 ± 1.06	− 30%	< 0.001
Mediwrap	N	N	N	1.3283 ± 0.0018	− 1.13%	0.006	23.83 ± 2.23	− 8%	0.261	71.40 ± 4.33	− 49%	< 0.001

*N* none; *M* minor; *S* significant. Radiation doses presented as mean value ± standard deviation

Removal of the backboard from under the phantom increased the absorbed dose by 20.33% (*p* < 0.001) but decreased the scattered dose by 30% (*p* = 0.003) and 55% (*p* < 0.001) in the anesthesiologist’s and operator’s locations.

### Image quality assessment

**Fig. 3:**
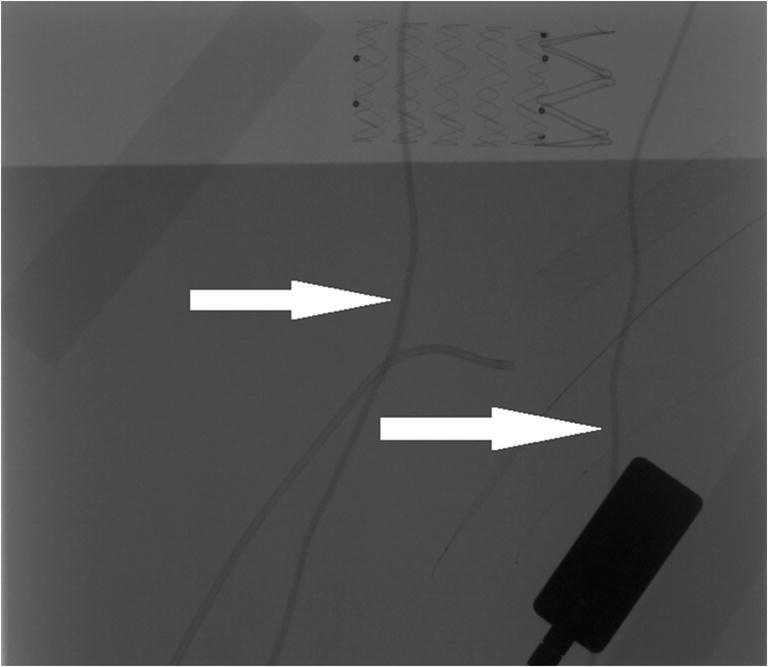
Linear artifacts caused by Helios cover (arrows)

**Fig. 4:**
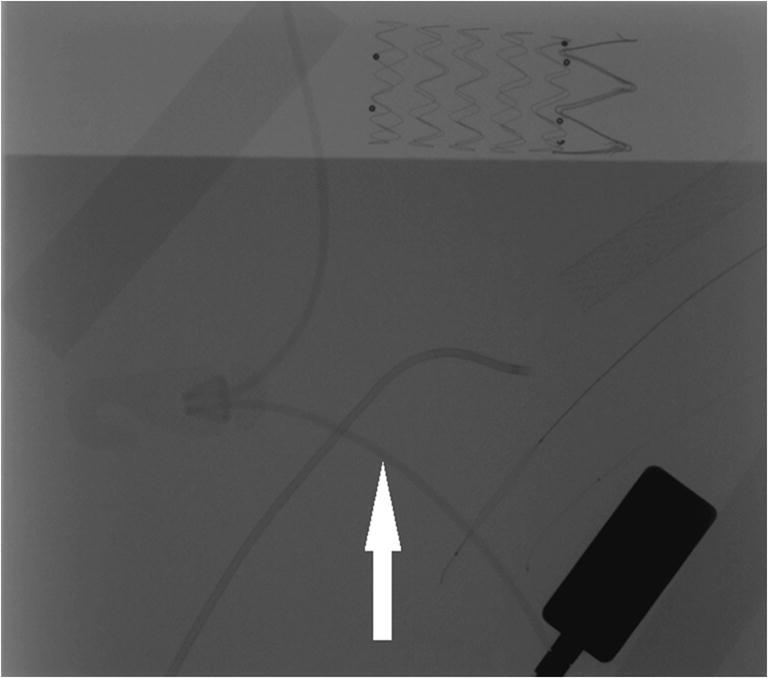
Linear artifacts from LESS clasp strings (arrow)

**Fig. 5:**
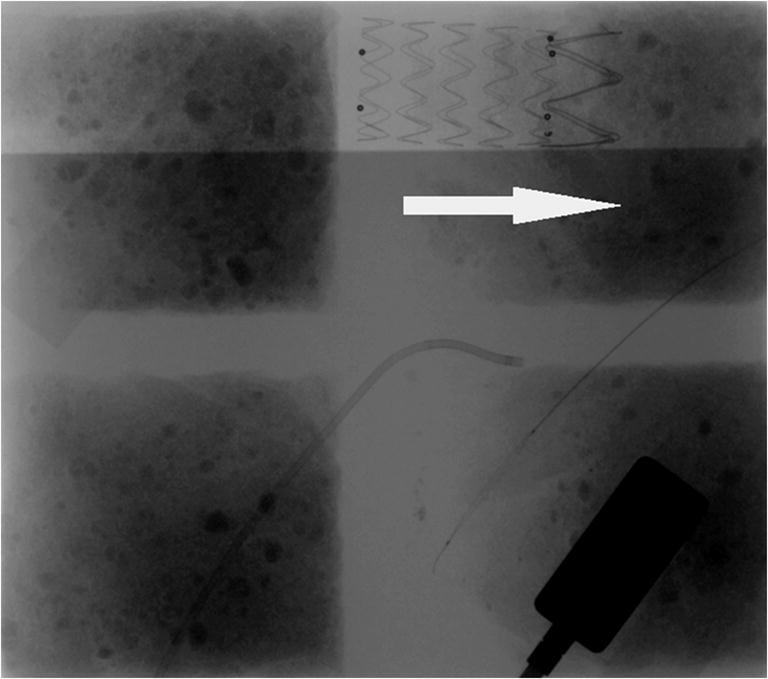
The stent hidden behind artifacts caused by Ready Heat blanket (arrow)

Most of the assessed systems did not cause visible artifacts. However, both LESS and Helios covers caused linear artifacts that did not veil endovascular equipment (Figs. 3 and 4). Only the Ready Heat blanket induced massive artifacts that obscured stents and catheters (Fig. 5). The artifacts’ assessment by the three physicians (A, B, and C) has been summarized in Table 1.

The resolution of images did not worsen. In all images without significant artifacts, catheter markers remained visible, including that which was the smallest.

## Discussion

None of the insulating covers tested caused significant artifacts in the fluoroscopy. Only one of the two tested warming systems (Ready Heat) induced large artifacts that hindered visual control of the endovascular procedure. As suspected, these were due to metallic objects contained in the chemical warming pads. Hence, the active warming of patients may be provided with forced-air warming devices which do not cause artifacts.

Radio-opaque elements of medical equipment may cause artifacts and, subsequently, affect the interpretation of radiographs [11, 12]. The linear artifacts caused by LESS and Helios covers may be misinterpreted, in some circumstances, as catheters. However, as similar linear elements such as ECG wires are usually present in the fluoroscopy, an experienced operator should be familiar with them.

The forced-air warming cover (Warm Touch), as one of two warming devices tested in our study, is radiolucent and does not cause artifacts. This warming method has been proven to be effective during elective aortic surgery [13]. However, patients that undergo emergency aortic repair may remain hypothermic when warmed without appropriate thermal insulation [4, 14]. The assessment of artifacts caused by insulating and warming systems on CT brought similar results. Self-heating pads (e.g., Ready Heat) induced significant artifacts which may hinder the image analysis, while forced-air warming cover appeared radiolucent [10]. Several insulating covers tested in that study did not cause artifacts apart from ties of LESS Bag.

The medical staff is exposed to scattered radiation during fluoroscopy. In our study, most of the insulating covers decreased both absorbed and scattered dose in the fluoroscopy. The dose depends on the distance from radiation source which has also been shown in other studies. Van Rappard et al. and de Ruiter et al. have demonstrated that the first operator absorbs a higher dose than other staff members in an operating room, which is similar to our results [15, 16]. Although radiation values in the anesthesiologist location were lower than those in the operator, in every wrapping scenario, their proportion was not constant. Hence, the distribution of the scattered radiation in the operating room was not homogeneous, while its intensity depended not only on the distance from an X-ray axis.

The highest increase of both absorbed and scattered doses was observed when a self-heating blanket (Ready Heat) was used. Probably, the active ingredients of the warming pad, namely carbon and iron particles, led to an increase in lamp current. A similar effect was reported by Sensakovic et al. in a study assessing the infant warming mattress filled with sodium acetate in computed tomography. This warming device increased tube current and, subsequently, the radiation dose by almost 30% [17].

The proportion of absorbed doses with and without a spineboard in our study (0.83) is similar to that reported by Hemmes et al. (~ 0.85) [18]. Hence, immobilization devices reduce radiation absorbed by the patient alongside fracture stabilization. However, they increase the scattered radiation absorbed by medical staff.

### Limitations

There are some limitations in this study. The number of covers tested in our study is limited and may not reflect the equipment of some emergency services. In a real patient, insulating covers may be repositioned or folded in order to facilitate the vascular access and in this way change the radiation dose. Scattered dose was measured in the absence of medical staff due to ethical reasons. As the personnel and additional equipment may absorb or reflect radiation, the real distribution of scattered radiation may be different.

## Conclusions

In conclusion, thermal insulation applied pre-hospitally in trauma victims in order to prevent post-traumatic hypothermia can be maintained during emergency endovascular procedures. Most of the systems tested in this study, including metalized reflective foils, did not cause artifacts in fluoroscopy and reduced the radiation dose. However, self-heating chemical blankets should be removed and replaced with another warming system.
